# No effects of COVID-19 on the development of type 1 diabetes autoimmunity and no evidence of an increased frequency of SARS-CoV-2 antibodies in newly diagnosed type 1 diabetes patients relative to healthy subjects

**DOI:** 10.1007/s00592-023-02103-1

**Published:** 2023-05-12

**Authors:** Claudio Tiberti, Raffaella Nenna, Valeria Tromba, Tiziana Filardi, Laura Petrarca, Francesca Silvestri, Valeria Fassino, Monica Montuori, Enrica Mancino, Andrea Lenzi, Fabio Midulla, Francesco Costantino, Susanna Morano

**Affiliations:** 1 Department of Experimental Medicine, “Sapienza” University of Rome, Viale Regina Elena 324, 00161 Rome, Italy; 2 Department of Maternal, Infantile and Urological Sciences, “Sapienza” University of Rome, Viale Regina Elena 324, 00161 Rome, Italy

**Keywords:** COVID-19, SARS-CoV-2, SARS-CoV-2 antibodies, Type 1 diabetes, Autoimmunity

## Abstract

**Aims:**

To evaluate the relationship between SARS-CoV-2 infection and autoimmunity in type 1 diabetes (T1D) and SARS-CoV-2 antibodies frequency at diagnosis of T1D during pandemic.

**Methods:**

The presence of T1D-specific autoimmunity was evaluated in a cohort of 99 children and adolescents without diabetes that contracted SARS-CoV-2 infection. Moreover, the frequency of IgM- and IgG-SARS-CoV-2 antibodies was evaluated in 41 newly diagnosed T1D patients not yet vaccinated against SARS-CoV-2 disease, collected during the pandemic, compared to healthy subjects (CTRL).

**Results:**

None of the 99 patients that contracted SARS-CoV-2 infection during the pandemic period was found positive for T1D autoantibodies. The frequency of SARS-CoV-2 antibodies was not significantly different in patients newly diagnosed with T1D (12.2%), compared with CTRL (8.4%). Among SARS-CoV-2 antibody positive T1D patients, 80% were target of diabetes autoantibodies and 60% had another concomitant autoimmune disease. Among the CTRL subjects positive for SARS-CoV-2Abs (*n* = 10), none was found positive for T1D autoantibodies.

**Conclusions:**

The results of the present study do not confirm, at least in the short term, a role of COVID-19 as a potential trigger of T1D autoimmunity and do not provide evidence of an increased frequency of SARS-CoV-2 antibodies in newly diagnosed T1D patients in comparison with healthy population.

## Introduction

Autoimmune diabetes pathogenesis was often associated with viral infections [1, 2] and the increased number of children with newly diagnosed type 1 diabetes (T1D) reported during the COVID-19 infection [3] seems to suggest a possible association of T1D with SARS-CoV-2 disease. Angiotensin-converting enzyme 2 (ACE2) has been identified as the receptor for the coronavirus spike protein [4, 5]. This finding, and the detection of significant amounts of ACE2 in the endocrine pancreas [6], lead to the hypothesis that the dysregulation of ACE2 activity following COVID-19 infection could induce beta cell damage and new onset diabetes [7]. However, to date, no compelling evidence emerged to confirm a role of COVID-19 as a potential trigger of T1D, and it remains unclear whether there is a causal role of SARS-CoV-2 infection in a change of diabetes incidence. In particular, to our knowledge, no data are available on the effects of COVID-19 infection on the development of T1D autoimmunity and few, discordant information are available on SARS-CoV-2 antibody frequencies at diagnosis of T1D during pandemic, with percentages of positive patients ranging from 0 to 19% [3, 8–10]. Based on these considerations and to gain new insights on the relationship between COVID-19 and T1D, aims of our study were:

a) to evaluate the presence of T1D-specific autoimmunity in a cohort of children and adolescents without diabetes that contracted SARS-CoV-2 disease (CoV-2) during the pandemic period;

b) to evaluate, in children and adolescents at T1D diagnosis not yet vaccinated against SARS-CoV-2 disease, the frequency of IgM- and IgG-SARS-CoV-2 antibodies during the pandemic period, comparing the relative results to those found in a population of healthy subjects (CTRL). In addition, we compared the humoral T1D, celiac, thyroid, gastric and adrenal autoantibody patterns of CoV-2 antibody positive and negative T1D patients.

## Patients and methods

### Patients

All the sera analyzed in the present study (*n* = 259) were collected in the Departments of Maternal, Infantile and Urological Sciences and Experimental Medicine “Sapienza” University of Rome, Rome, Italy. In particular, CoV-2 patient sera were collected in the pandemic period February-April 2021, whereas T1D patients and CTRL subject sera were collected sequentially in the pandemic period 2020–2021. None of the individuals investigated in the study was vaccinated against SARS-CoV-2 disease at the moment of serum collection. Serum samples were subdivided as follows (see also Table 1): 99 CoV-2 sera from children and adolescent patients that contracted SARS-CoV-2 disease during the pandemic [50 females, 49 males; median age 9.4 years; age range 0.4–17.4 years; 82.8% symptomatic during COVID-19, 94.9% positive for SARS-CoV-2 antibodies, the interval between the first oropharyngeal swab and the medical examination was of 114 days (range 34–332 days)]. Among the CoV-2 patients, 22.2% were relatives of patients with endocrine pathologies, 2.0% were celiac at gluten-free diet, and none had type 1 diabetes mellitus and was relative of a T1D patient. Forty-one T1D patient sera at disease diagnosis (20 females, 21 males; median age 9.6 years; age range 1–16 years). 119 CTRL subject sera (65 females, 54 males; median age 8.8 years; age range 1.1–15,9 years). The study was approved by the local Ethics Committee and conducted in accordance with the principles expressed in the Declaration of Helsinki. All subjects provided written informed consent before the enrollment.

**Table 1:** Patients and controls recruited for the study

	Type 1 diabetes	Controls	COVID-19
Number	41	119	99
Median age (years)	9.6	8.8	9.4
Age range (yrs)	1–16	1.1–15.9	0.4–17.4
Females/Males	20/21	65/54	50/49

### Methods

#### SARS-CoV-2 IgM and IgG detection

IgM and IgG antibodies to SARS-CoV-2 were detected by using on the ARCHITECT i System (Abbott) the chemiluminescent microparticle SARS-CoV-2 IgM (code SR87, Abbott) and IgG (code SR86, Abbott) immunoassays, respectively. Both assays were designed to detect IgM or IgG antibodies to the nucleocapsid protein of SARS-CoV-2. The chemiluminescent reaction was measured as a relative light unit (RLU). There is a direct relationship between the amount of IgM or IgG antibodies to SARS-CoV-2 in the serum sample and the RLU detected by the system optics. The presence or absence of IgM or IgG antibodies to SARS-CoV-2 in the serum sample was determined by comparing the RLU in the reaction (S) to the RLU of relative calibrator (C). Serum samples were considered SARS-CoV-2-IgM and -IgG antibody positive if the Ab-indexes (S/C) were ≥ 1.0 and ≥ 1.4, respectively. All the T1D patients found positive for IgM and/or IgG antibodies to the nucleocapsid protein of SARS-CoV-2, were analyzed also for presence of IgG antibodies directed against the subunit 1 of the spike protein receptor-binding domain of the SARS-CoV-2 (ARCHITECT i System, chemiluminescent microparticle SARS-CoV-2 IgGII Quant immunoassay, code 6S60-22, Abbott). Serum samples were considered SARS-CoV-2-IgII antibody positive if the Ab-indexes (S/C) were ≥ 50 AU/mL.

#### T1D-specific autoimmunity detection

The immune response directed against four pancreatic islet proteins specific to autoimmune diabetes [insulin (Ins), glutamic acid decarboxylase (GAD), tyrosine phosphatase 2 _(605–979)_ (IA-2ic) and islet beta-cell zinc cation efflux transporter (ZnT8)] was measured by using a single combined fluid-phase radioimmunoprecipitation assay based on the detection of the four autoantibodies in a single assay (MAA) [11]. MAA showed 92% sensitivity and 99% specificity in the Diabetes Antibody Standardization Program (DASP) held in 2010. All the patients found MAA autoantibody positive were subsequently analyzed for single T1D-specific autoantibodies [11].

#### IgA-anti-transglutaminase Ab detection (IgA-tTGAbs)

Serum IgA-tTGAbs were detected by a fluid-phase radioimmunoprecipitation method using the full-length human tTG cDNA transcribed and translated in vitro in the presence of 35-S methionine [12]. This assay was reported to be the most sensitive and specific assay in the First International Transglutaminase Autoantibody workshop for celiac disease [13].

#### Thyroid peroxidase Abs (TPO-Abs)

TPO-Abs were measured by using a commercially available anti-TPO Chemiluminescent Microparticle Immunoassays (CMIA), (Architect System, Abbott Diagnostic Division, USA).

#### Gastric parietal cell antibodies (APC Abs)

APC Abs were analyzed by a commercially available ELISA (Axa Diagnostics, Pomezia Italy).

#### 21-hydroxylase antibodies (21(OH)-Abs)

Were detected by a radioimmunoprecipitation assay using recombinant human 21-OH radiolabeled with [35S], as previously described [13]. The 21-OH assay obtained 94.2% sensitivity and 100% specificity at the Standardization program for determination of 21OHAb held in 2011 [14].

## Results

### T1D autoantibodies in CoV-2 patients

None of the 99 patients that contracted SARS-CoV-2 disease during the pandemic period was found positive for type 1 diabetes autoantibodies.

### IgM- and IgG-SARS-CoV-2 antibodies in T1D and CTRL patients

Results of T1D patients are shown in Table 2. Of 41 T1D patients at diagnosis 5 (12.2%) were found positive for nucleocapsid SARS-CoV-2 Abs, with a frequency not significantly different from that found in CTRL subjects (10/119, 8.4%). Four of these patients were females (80%). One T1D patient was found positive for both nucleocapsid SARS-CoV-2 IgM and IgG, 2 for SARS-CoV-2 IgM and 2 for SARS-CoV-2 IgG. All of the 5 T1D patients were positive also for IgG antibodies directed against the subunit 1 of the spike protein receptor-binding domain of the SARS-CoV-2. The remaining 36 T1D patients, negative for nucleocapsid SARS-CoV-2 Abs, were negative also for spike protein SARS-CoV-2 Abs. T1D patients positive for nucleocapsid SARS-CoV-2 Abs had higher, but not statistically different median age, age range, HbA1c, C-peptide, DKA-grade and BMI mean values in comparison with T1D patients negative for nucleocapsid SARS-CoV-2 Abs. Only one (20%) of the 5 T1D patients positive for nucleocapsid SARS-CoV-2 Abs was negative for T1D autoantibodies, whereas the residual 4 (80%) were positive for ≥ 2 autoantibodies. Of note, all of these 4 patients were positive for insulin autoantibodies, significant more frequently than T1D patients negative for nucleocapsid SARS-CoV-2 Abs (27.8%, *p* = 0.039). Among the CTRL subjects positive for nucleocapsid SARS-CoV-2 Abs (70% females), one was positive for both nucleocapsid SARS-CoV-2 IgM and IgG, 2 for SARS-CoV-2 IgM and 7 for SARS-CoV-2 IgG. None of the CTRL subjects was positive for T1D autoantibodies. Table 3 shows the characteristics of the 5 patients that at T1D diagnosis were found positive for SARS-CoV-2 antibodies. Two of them had concomitant celiac disease and one thyroid disease.

**Table 2:** Clinical characteristics and laboratory parameters of T1D patients

	T1D	CoV-2 Ab-positive T1D	CoV-2 Ab-negative T1D
N°	41	5	36
Females/males	20/21	4/1	16/20
Median age (yrs)	9.5	12.0	8.9
Age range (yrs)	1.4–15.9	10.6–13.3	1.4 – 15.9
FPG mean (mg/dL)	373.2 (112–709)	298.4 (115–435)	383.6 (112–709)
HbA1c—mean (range) (%) mmol/mol	10.9 (5.9–13.8) 96 (41–127)	11.5 (9.1–15) 102 (76–140)	10.7 (5.9–13.8) 93 (41–127)
DKA (%)	11 (26.8%)	2 (40%)	9 (25%)
DKA-grade mean	1.82	2.0	1.78
BMI mean (Kg/m^2^)	17.7 (13–30.8)	19.1 (14.8–23.8)	17.5 (13–30.8)
C-peptide mean (ng/ml)	0.60 (0.01–3.03)	1.12 (0.25–3.03)	0.55 (0.01–2.42)
n° T1D Abs (mean)	2.07	2.4	2.03
GAD Abs (%)	33 (80.5%)	3 (60%)	30 (83.3%)
Ins Abs (%)	14 (34.1%)	4 (80%)*	10 (27.8%)
IA-2 Abs (%)	27 (65.9%)	3 (60%)	30 (83.3%)
ZnT8 Abs (%)	14 (34.1%)	2 (40%)	12 (33.3%)
No T1D Abs (%)	4 (9.8%)	1 (20%)	3 (8.3%)
IgA-anti-transglutaminase Abs (%)	11 (26.8%)	1 (20%)	10 (27.8%)
Thyroid Abs (TPO/TG Abs) (%)	8 (19.5%)	1 (20%)	7 (19.4%)
Gastric Parietal cell Abs (APCA) (%)	6 (14.6%)	0 (0%)	6 (16.7%)
21(OH) Abs (%)	11 (2.4%)	0 (0%)	1 (2.8%)
No organ-specific Abs (T1D excluded) (%)	22 (53.7%)	3 (60%)	19 (52.8%)

*p = 0.039 vs CoV-2 Ab-negative T1D

**Table 3:** Characteristics of T1D patients positive for SARS-CoV-2 antibodies

Patient	CoV2 Abs IgM	CoV2 Abs IgG	Sex	Age (yrs)	T1D first degree relatives	Glycemia Mg/dL	Ketoacidosis	HbA1c (%)	C-peptide (ng/ml)	T1D Abs GAD/AAI/IA-2/ZnT8	tTG Abs	TPO-Abs	APC Abs	21-OH Abs	Other features at T1D diagnosis
1	** + **	** + **	F	10.8	father	278	no	11.8	0.25	GAD/AAI/IA-2/ZnT8	**−**	**−**	**−**	**−**	**−**
2	**−**	** + **	M	13.3	no	230	yes	15.0	0.87	AAI/IA-2/ZnT8	** + **	**−**	**−**	**−**	Celiac disease
3	**−**	** + **	F	12.0	no	115	no	9.1	3.03	GAD/AAI	**−**	** + **	**−**	**−**	Thyroiditis
4	** + **	**−**	F	10.6	no	275	no	9.8	0.56	GAD/AAI/IC	**−**	**−**	**−**	**−**	**−**
5	** + **	**−**	F	14.6	mother	255	no	11.6	0.89	No Abs	**−**	**−**	**−**	**−**	Celiac disease

*T1D* Type 1 diabetes; *GAD* Glutamic acid decarboxylase; *IA-2* Tyrosine phosphatase 2; *ZnT8* Islet beta-cell zinc cation efflux transporter; *AAI* Insulin autoantibodies; *tTG* Transglutaminase; *TPO* Thyroid peroxidase; *APC* Parietal cell; *21(OH)* 21-hydroxylase

## Discussion

No data are available so far on the effects of COVID-19 infection on the development of T1D autoimmunity. In the present study, by analyzing a cohort of children and adolescents without diabetes that contracted SARS-CoV-2 disease during the pandemic period February-April 2021, we found that COVID-19 infection was not able to induce, in a mean time interval of about four months from the first SARS-CoV-2 positive oropharyngeal swab, the development of T1D-related autoimmunity. Our data appear to be in contrast with the findings reported for viral infections by Löonrot et al. in 2017 [2], that were however relative to patients genetically at risk to develop T1D analyzed 0–9 months prior to the development of islet autoimmunity. A possible explanation for these discordant results is that the impact of COVID-19 infection might be different in distinct categories of subjects, likely with more pronounced effects in individuals at risk to develop type 1 diabetes. The lack of T1D-related autoimmunity in our cohort of SARS-CoV-2 patients, which must obviously be confirmed in follow-up studies extended over a longer period of time, seems to be in contrast with the hypothesis that the dysregulation of ACE2 activity following COVID-19 infection could induce beta-cell damage and new onset diabetes [4], at least in the time window that we investigated, ranging from 1 to 11 months from the first SARS-CoV-2 positive oropharyngeal swab. Our data seem to be consistent with the results of Coate et al. [15] that detected ACE2 expression in in pancreatic microvasculature and ductal cells but not in beta cells, suggesting a low probability that ACE2 is able to mediate a direct beta-cell cytotoxicity. A second aim of our study was to evaluate in children and adolescents at T1D diagnosis, not yet vaccinated against SARS-CoV-2 disease, the frequency of CoV-2 antibodies during the pandemic period, comparing the relative results to those found in a population of healthy subjects collected in the same period of time (2020–2021). Of note, the analysis of patients not vaccinated for COVID-19 is a feature that will be likely not proper of future studies on this topic, including those relative to follow-up of patients, that will necessarily have to take in account that many subjects underwent one or more doses of vaccine. We found that 12.2% of T1D patients at diagnosis were positive for SARS-CoV-2 antibodies. This consistent percentage of patients was however not significantly different respect to that found in healthy controls without diabetes collected in the same pandemic period of time (8.4%). Our data confirm the results of other studies [3, 9, 10], where no significantly different SARS-CoV-2 antibody frequencies were found between T1D patients and healthy controls. Salmi et al. [10], with a baseline prevalence of their own region of 0.6%, did not detect SARS-CoV-2 antibodies in any of the 20 newly diagnosed T1D children recruited in the pandemic period April–October 2020. Jia et al. detected 0.8% SARS-CoV-2 antibodies in T1D patients and 2.8% of controls in samples collected between January and October 2020 [9]. Ata et al. [8] found SARS-CoV-2 antibodies in 8.7% of T1D patients and 10% of controls. If it is evident that all these studies were concordant to exclude a higher percent of SARS-CoV-2 antibody positive individuals in T1D patients compared to healthy population, it is equally evident that a wide range of autoantibody frequencies was found from one study to another for both diabetes patients and controls. It is likely that these different frequencies might reflect, in the various cohorts of patients investigated, different time phases of the pandemic. The evaluation, in our study, of T1D-specific humoral autoimmunity in SARS-CoV-2 antibody positive diabetes patients and controls provided other interesting information. We found that 80% of the T1D patients at disease diagnosis were target of at least two diabetes autoantibodies, suggesting that the manifestations of T1D following or concomitant with SARS-CoV-2 infection occur not only in absence of the autoantibodies typical for T1D, as supposed in the report case of Hollstein et al. in a 19-year-old male patient [16]. Considering that our cohort of diabetes patients had an age range comprised between 1 and 16 years, it will be interesting to assess in future studies, if diabetes-specific immunoreactivity may occur also in adult SARS-CoV-2 antibody positive T1D patients. Of note, all the T1D patients identified as SARS-CoV-2 autoantibody positive in our study were in school age, between 10.6 and 14.6 years, this suggesting a possible higher risk to contract COVID-19 infection among students. Another interesting observation emerging from our study was that all the SARS-CoV-2 autoantibody positive T1D patients with diabetes immunoreactivity were positive for insulin autoantibodies, significantly more frequently than T1D patients negative for nucleocapsid SARS-CoV-2 Abs. If confirmed in a larger number of patients, this finding could be of value in future studies aimed to understand whether COVID-19 could be involved in the pathogenesis of beta-cell destruction in these patients. Very little is known about the impact of COVID-19 on patients with multiple autoimmune diseases, especially in children. In our study, 3 out of 5 SARS-CoV-2 autoantibody positive T1D patients (60%) also had another concomitant autoimmune disease (*n* = 2 celiac disease and 1 thyroid disease), leading to the question if patients with multiple autoimmune diseases are more susceptible to virus infection. Among the CTRL subjects positive for nucleocapsid SARS-CoV-2Abs, none was found positive for T1D autoantibodies. This finding, in addition to the lack of diabetes-specific immunoreactivities in our cohort of children and adolescents without diabetes that contracted SARS-CoV-2 disease during the pandemic period February-April 2021, suggests that the risk of developing T1D immunoreactivity following COVID-19 infection, at least in the time windows investigated and in subjects not at risk to develop T1D, is practically absent. Of course, this observation does not exclude that other viral mechanisms different from the development of humoral autoimmunity could have a potential role in an eventual pathogenesis of T1D. The relatively recent worldwide appearance of the SARS-CoV-2 virus, leading to a still insufficient knowledge of mechanisms that regulate its function, makes it difficult to formulate a convincing hypothesis about how SARS-CoV-2 virus might be potentially associated with the pathogenesis of T1D. If these mechanisms exist, they might be of biochemical origin (as suggested by the studies demonstrating the dysregulation of ACE2 activity following COVID-19 infection) and the eventual appearance of a humoral diabetes-specific immune response might be only a secondary effect of the viral action, although important to identify the COVID-19 patients susceptible to develop T1D. In conclusion, the results of the present study, despite not conclusive, do not confirm, at least in the short term, a role of COVID-19 as a potential trigger of T1D autoimmunity and do not provide evidence of an increased frequency of SARS-CoV-2 antibodies in newly diagnosed T1D patients in comparison to healthy population. Additional studies are warranted to explore the exact time course and effects of SARS-CoV-2 on pancreatic islet cells.

## Data Availability

The data that support the findings of this study are available from the corresponding author upon reasonable request.
