# Correction to: No effects of COVID‑19 on the development of type 1 diabetes autoimmunity and no evidence of an increased frequency of SARS‑CoV‑2 antibodies in newly diagnosed type 1 diabetes patients relative to healthy subjects

**DOI:** 10.1007/s00592-023-02152-6

**Published:** 2023-07-20

**Authors:** Claudio Tiberti, Raffaella Nenna, Valeria Tromba, Tiziana Filardi, Laura Petrarca, Francesca Silvestri, Valeria Fassino, Monica Montuori, Enrica Mancino, Andrea Lenzi, Fabio Midulla, Francesco Costantino, Susanna Morano

**Affiliations:** 1 Department of Experimental Medicine, “Sapienza” University of Rome, Viale Regina Elena 324, 00161 Rome, Italy; 2 Department of Maternal, Infantile and Urological Sciences, “Sapienza” University of Rome, Viale Regina Elena 324, 00161 Rome, Italy

**Correction to: Acta Diabetologica** 10.1007/s00592-023-02103-1

Given name and family name of authors published incorrect in the original publication. The author names now corrected here.

The original article has been corrected.

